# Standard or hypofractionated radiotherapy in the postoperative treatment of breast cancer: a retrospective analysis of acute skin toxicity and dose inhomogeneities

**DOI:** 10.1186/1471-2407-13-230

**Published:** 2013-05-07

**Authors:** Grazia Tortorelli, Luana Di Murro, Rosaria Barbarino, Sara Cicchetti, Daniela di Cristino, Maria Daniela Falco, Dahlia Fedele, Gianluca Ingrosso, Dania Janniello, Pasquale Morelli, Alessandra Murgia, Elisabetta Ponti, Sara Terenzi, Barbara Tolu, Riccardo Santoni

**Affiliations:** 1 Department of Diagnostic Imaging, Molecular Imaging, Interventional Radiology and Radiotherapy, Tor Vergata University General Hospital, Viale Oxford 81, Rome 00133, Italy

## Abstract

**Background:**

To identify predictive factors of radiation-induced skin toxicity in breast cancer patients by the analysis of dosimetric and clinical factors.

**Methods:**

339 patients treated between January 2007 and December 2010 are included in the present analysis. Whole breast irradiation was delivered with Conventional Fractionation (CF) (50Gy, 2.0/day, 25 fractions) and moderate Hypofractionated Schedule (HS) (44Gy, 2.75Gy/day, 16 fractions) followed by tumour bed boost. The impact of patient clinical features, systemic treatments and, in particular, dose inhomogeneities on the occurrence of different levels of skin reaction has been retrospectively evaluated.

**Results:**

G2 and G3 acute skin toxicity were 42% and 13% in CF patients and 30% and 7.5% in HS patients respectively. The retrieval and revaluation of 200 treatment plans showed a strong correlation between areas close to the skin surface, with inhomogeneities >107% of the prescribed dose, and the desquamation areas as described in the clinical records.

**Conclusions:**

In our experience dose inhomogeneity underneath G2 – G3 skin reactions seems to be the most important predictor for acute skin damage and in these patients more complex treatment techniques should be considered to avoid skin damage. Genetic polymorphisms too have to be investigated as possible promising candidates for predicting acute skin reactions.

## Background

Radiation Therapy (RT) has gained an established role in the treatment of breast cancer either as chest wall irradiation for high risk patients after modified radical mastectomy, or as whole breast irradiation for patients after a breast conserving surgery (BCS). The challenge now is to minimise the morbidity caused by treatment without losing its efficacy and to select patients at risk of developing skin toxicity who deserve more complex treatment techniques able to reduce this problem. Acute and chronic toxicities have been reported in patients after breast or chest wall RT [1,2]. Postoperative RT for breast cancer patients is delivered using conventional tangential fields with dose inhomogeneities resulting in an excess irradiation of breast tissue. Three-dimensional conformal Radiation Therapy (3D-CRT), IMRT and Tomotherapy are associated with relatively lower risks of toxicity compared with 2D technique [3,4]. Skin toxicity can lead to temporary or permanent cessation of treatment, pain, occasionally systemic infection, and may cause permanent skin changes. This problem may probably be reduced improving dose conformity and dose homogeneity within the irradiated area and in close proximity of the skin surface in spite of the fact that complex techniques as IMRT are time consuming and more expensive. Few studies [5] have addressed this item, but off-axis dose inhomogeneities have rarely been considered although it has been suggested by some Authors [6].

With the limits of retrospective studies, some data have suggested that dose inhomogeneties (V > 107%) was a significant predictor of RT-induced skin toxicity on the occurrence of severe skin reactions [7,8].

The aim of our analysis is to try to relate “hot spot” volumes, sites and amount of dose inhomogeneities to skin toxicity in a set of patients who underwent 3D conformal irradiation whose 3D treatment plans were retrieved and revaluated to calculate the volumes of Planning Target Volume and Treated Volume receiving more than 107% of the prescribed dose.

## Methods

### Characteristics of patients and data collection

Between January 2007 and December 2010, 339 evaluable patients for the present analysis, with histological confirmed early breast cancer (pT1-2, pN0-1), were referred, for post-operative treatment after breast conservative surgery, to our Radiation Therapy Unit. The main clinical features of these patients and of the tumours are reported in the Table 1. All of the patients provided an informed consent for breast irradiation. Patients who received prior breast irradiation, presented bilateral breast cancer, affected by seromas, wound infection, connective tissue disorders were excluded by the present evaluation.

**Table 1:** Main features of the 339 reported patients

	**Total patients (%)**	**CF Patients (%)**	**HS Patients (%)**	**P Value**
No.	339	141 (41.59)	198 (58.41)	
Mean age (range)	60 (22–86)	52 (22–79)	62.5 (38–86)	*<0.001*
Histology				NS
ILC	30 (8.8)	12 (8.5)	18 (9.1)	
IDC	299 (88.2)	126 (89.4)	173 (87.4)	
Intraductal	10 (3)	3 (21)	7 (3.5)	
T stage				NS
T1	293 (86.4)	121 (85.8)	172 (86.7)	
T2	46 (13.6)	20 (14.2)	26 (13.3)	
N stage				NS
Nx	21 (6,3)	8 (5,7)	13 (6,5)	
N0	251 (74)	93 (65.9)	158 (79.8)	
N1	67 (19.7)	32 (22.6)	35 (17.6)	
Grading (Bloom Richardson’s scale)				NS
G1	60 (17.7)	22 (15.6)	38 (19.2)	
G2	131 (38.6)	58 (41.1)	73 (36.8)	
G3	79 (23.3)	36 (25.5)	43 (21.7)	
NA	69 (20.4)	25 (17.8)	44 (22.3)	
Surgical margins				NS
Negative	295 (87)	124 (87.9)	171 (86.4)	
Positive	5 (1.5)	2 (1.4)	3 (1.5)	
Close (< 2 mm)	27 (8)	10 (7.1)	17 (8.6)	
Tangents	12 (3.5)	5 (3.6)	7 (3.5)	
Breast Volume				
Average cc	718.7	684	725.4	NS
(range)	(188.6-2036.7)	(188.6-1899.9)	(193.3-2036.7)	
Chemotherapy				
Yes	126 (37.2)	68 (48.2%)	58 (29.3%)	
No	211 (62.2%)	72 (51.1%)	139 (70.2%)	0.008
NA	2 (0.6%)	1 (0.7%)	1 (0.5%)	
Hormone therapy				
Yes	247 (72.8)	101 (71.6)	146 (73.7)	NS
No	74 (21.8)	34 (24.1)	40 (20,2)	
NA	18 (5.4)	6 (4.3)	12 (6.1)	
Trastuzumab				
Yes	17 (5)	7 (5)	10 (5)	NS
No	327 (95)	134 (95)	188 (95)	

CF: Conventional Fractionation; HS**:** Hypofractionated Schedule; ILC: infiltrating lobular carcinoma; IDC: infiltrating ductal carcinoma; NA: not available; NS: not significant (P>0.05).

All of these patients underwent a clinical examination before irradiation, weekly during the treatment course and one week after the end of treatment, every month for three months and at regular time intervals (every three months) afterwards. One hundred and twenty six patients received adjuvant chemotherapy after surgery and before RT. Hormone therapy (tamoxifen or aromatase inhibitors) were prescribed to 247 patients. Written informed consent was obtained from the patients for the purpose of this report as well as it concerns any accompanying image. Our Institutional Ethic Committee (Comitato Etico Indipendente, Fondazione Policlinico Tor Vergata, Roma) approved this study (protocol number 104/12).

### Total dose and fractionation

Whole breast irradiation was delivered with Conventional Fractionation (CF) in 141 patients (50 Gy, 2 Gy/day in 25 fractions) and moderate Hypofractionated Schedule (HS) in 198 ones (44 Gy, 2.75 Gy/day in 16 fractions) followed by an electron tumour bed boost (10–16 Gy, 2 Gy/day in 5–8 fractions or 9–15 Gy, 3 Gy/day in 3–5 fractions).

### Biological equivalent dose

The biologically effective doses (BED) were calculated assuming 4 Gy α/β ratio (tumour control), 10 Gy (acute responding normal tissues) [9].

Differences in fractionation and overall treatment time were taken into account using the following formula: BED = ***(n·d) (1+d/(α/β))-(ln2/(α·Tpk )·(T-Tk)***.

Only for the CF and for tumour control the time factor (2nd addendum in the above equation) has been taken into account. BEDs for HS was calculated assuming zero as time factor [9]. Description of the parameters used in the above formula are detailed in the Appendix.

### Localization and planning

Planning CT scans (5 mm slice thickness) from the level of the larynx to the upper abdomen, including both lungs, were obtained in the supine or prone position depending on patient tolerance and anatomy. Women in supine set-up were positioned using a “wing-board” (BIONIX Development Corporation, Toledo, Ohio) with both arms raised above their head. Prone patients were positioned using a commercially available immobilization device (CIVCO, Orange City, USA).

CT data were transferred to Precise Plan® (Elekta, Crawley, United Kingdom). Clinical Target Volume (CTV) included whole breast tissue and was expanded by 10 mm, but within 5 mm from the skin surface, to create the planned target volume (PTV). Organs at Risk (OARs), lung and heart, were contoured according to the 50 and 62 International Commission on Radiations Units and Measurements Reports Recommendations (ICRU) [10,11]. 3D-CRT plans with opposing tangential beams were planned to cover the PTV and a multi-leaf collimator (MLC) was used, when necessary, to minimize the dose delivered to the OARs. Beam energy was 6 MV; mixed energies (6 MV-10 MV) were occasionally used in larger breasts. Wedges were used in almost all of the patients to provide a homogenous PTV dose (95% of 50 Gy and 44 Gy for the CF and HS groups respectively). For each patient, dose-volume histograms (DVHs) for the target and OARs were obtained. Boost doses were delivering using a 6–12 MeV electron fields. All patients were treated using the Elekta Precise® Accelerator (Elekta, Crawley, United Kingdom).

### Assessment of acute skin toxicity

Toxicity was described as the maximum reported acute toxicity, either during or after completion of RT, as described in the clinical records. Clinically evident skin reactions (G2 – G3) were assessed using the RTOG Acute Morbidity Scale defining grade G2 as tender or bright erythema, patchy moist desquamation/moderate oedema, and grade G3 as confluent moist desquamation, other than skin folds, pitting oedema. Grade G0 (no reaction) and Grade G1 (faint reaction) were considered in a common category as concerns the statistical evaluation of the results.

### Dosimetry data collection

Our analysis took origin from the empirical observation of the occurrence of G3 skin reactions in unexpected patients who did not show clinically detectable features predicting adverse effects.

**Figure 1:**
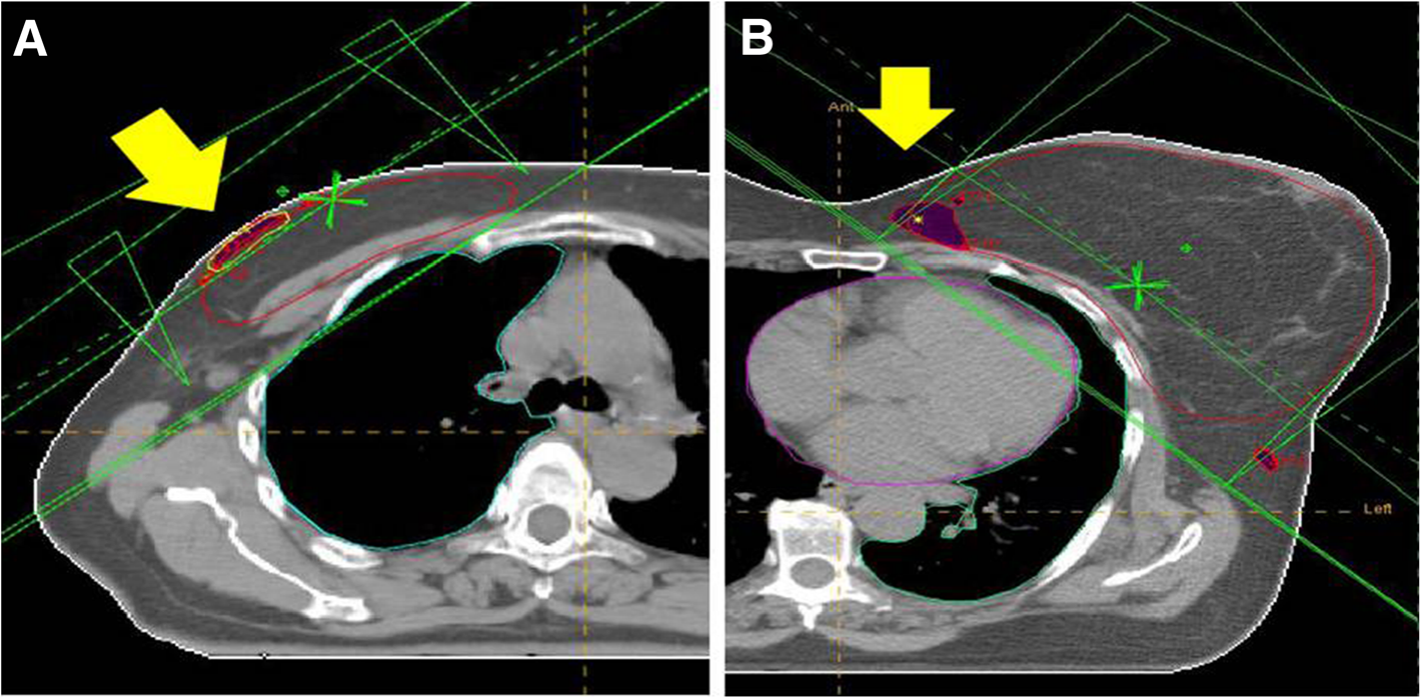
**Dose distribution in different breasts with and without moist desquamation. 1A** Treatment plan of a patient developing moist dry desquamation of the external upper quadrant. The V > 107% is located close to the skin surface (yellow arrow). **1B** - Treatment plan of a patient with large breast not developing skin toxicity. The V > 107% is located deeply in the breast parenchyma (yellow arrow).

Revaluating the treatment plans of these patients we observed inhomogeneities of dose distribution which were related to the higher skin toxicities appearing before or after the end of treatment. Following this observation, treatment plans were retrieved and revaluated and a strong correlation was found between areas close to the skin surface, with an inhomogeneity > 107% of the prescribed dose, and the desquamation areas as described in the clinical records. This quantitative dose-volume analysis was performed in 200 patients representing more than 60% of all the patients with any toxicity grade. We contoured, on each scan from the CT planning within and outside the target, the Regions of Interest (ROIs) corresponding to the areas receiving a dose in excessive > 107% of the prescribed one (> 53.5 Gy for CF and > 47.1 Gy for HS) which were named V > 107 (cc) (Figure 1). The maximum doses, over 107% of the prescribed ones, were also recorded. Breast volume was estimated using volumetric measurements of the planning target volume (PTV). Dosimetry data are reported in Table 2.

**Table 2:** Treatment and dosimetric characteristics in 200 patients whose plans were revaluated

**CF: 79/141 (56%)**	**HF: 121/198 (61,1%)**
***PTV (Gy)***	***PTV (Gy)***
Mean Dose: 50,17	Mean Dose: 44,16
Median Dose: 50,30	Median Dose: 44,39
***V > 107% (cc)***	***V > 107% (cc)***
Mean Volume 10	Mean Volume 5.7
Median Volume 9	Median Volume 3
**Mean Maximum Dose (Gy)** 55,84	**Mean Maximum Dose(Gy)** 49,20

CF: Conventional Fractionation; HS**:** Hypofractionated Schedule.

### “In vivo” measurements

During the first treatment sessions we used the OneDosePlusTM MOSFET based as a dosimetric system for “in vivo” measurements in order to measure the dose actually delivered and to test the entire treatment procedure. Each MOSFET dosimeter was attached to the patient’s skin with its build-up cap area at the projection of the isocenter and as perpendicular as possible to the beam central axis. These doses were compared with those calculated with Precise Plan. For each patient, the measurements were performed using one MOSFET for each field of the treatment plan (both medial and lateral fields). We calculated an Action Threshold (AT), defined as the maximum acceptable discrepancy between the dose measured with the detector and the dose calculated with the TPS for each single field from phantom data [12]. The AT was ± 5% within 2 Standard Deviations.

### Statistical analysis

The χ2 and Mann–Whitney tests were used to compare acute skin toxicity between different sample groups and to analyze associations between acute toxicity, dosimetric parameters and clinical characteristics. Multivariate analysis to independently predict the risk of acute skin toxicity development was performed using binary logistic regression. Statistical significance was assumed at p < 0.05; data were processed using the Statistical Package of Social Sciences (SPSS) Version 17.0.

## Results

Acute toxicity rates (Table 3) were found to be higher among the patients undergoing CF (81.6%) with respect to those treated with the moderate HS (62.6%) and the difference, which is statistically significant (p < 0.001), is not surprising.

**Table 3:** Frequency of any grade of acute skin toxicity between the two groups of patients

**Total patients 339**		**CF**		**HS**		**P value**
		**141**		**198**		
		**Patients**	**Toxicity**	**Patients**	**Toxicity**	
		**(No.)**	**grade**	**(No.)**	**grade**	
**Acute Toxicity**	239	115	G1: 52 (45,2%)	124	G1: 79 (63,5%)	**< 0.001**
					G2: 36 (30%)	
		*(81,6.%)*	G2: 48 (42%)	*(62,6%)*	G3: 9 (7,5%)	
			G3: 15 (13%)			

In particular G2 and G3 acute skin toxicities were registered in 42% (48/115) and 13% (15/115) among the patients who underwent irradiation with CF and in 30% (36/124) and 7.5% (9/124) in the HS group respectively. The onset of the peak skin toxicity, among the patients undergoing conventional fractionation, appeared during the last week of treatment (between session number 20 and 25) while, on the contrary, it appeared immediately after the end of treatment among those undergoing hypofractionated irradiation (around session number 16). For this reason the treatment was interrupted in 5 patients only and all of them belonged to the CF group, but in none of those undergoing HF treatment. Eighty percent of skin reactions occurred at the level of the inframammary fold and the remaining ones in the upper quadrants-axilla. G2-G3 skin reactions were always in close proximity of an inhomogeneity of dose distribution which was located under the skin surface. No skin reaction was diagnosed in all of the patients in whom dose inhomogeneity were deep in the breast tissue distant from the skin.

Systemic treatments and patient and treatment related factors of all of the 339 patients have been evaluated in a univariate analysis (Table 4). Only age and fractionation were found to be statistically significant as concerns the occurrence of acute skin reaction (p < 0.001) while systemic treatments and volume of the breast did not show any influence on the occurrence of acute adverse effects (p = 0.80, p = 0.66 and p = 0.072 respectively for chemotherapy, hormone treatment, breast volume). In our analysis other clinical factors such as smoking, hypertension, diabetes, were not significantly correlated with the development of acute skin reaction. No topic treatment was prescribed in any of these patients to prevent or modify skin reaction.

**Table 4:** Univariate analysis: predictive factors for G1-G2-G3 radiation–induced skin reactions (339 patients)

**Variables**	***p *value**
Chemotherapy	0.806
(yes vs no)	
Hormone Therapy	0.665
(yes vs no)	
Age	*< 0.001*
(≤ average vs > average)	
Breast Volume	0.072
(≤ average vs > average)	
Fractionation Schedule	*< 0.001*
(CF vs HS)	

CF: Conventional Fractionation; HS**:** Hypofractionated Schedule.

The treatment plans of 200 patients, including approximately 60% of the patients belonging to the HS and CF groups, were retrieved and revaluated and volumes receiving more than 107% (V > 107%) of the prescribed dose were defined in each single case. Half of these patients showed no skin reaction (G0) or a very faint reaction (G1), while the remaining belonged to the G2 – G3 groups.

At the univariate analysis, as summarised in Table 5, younger age at diagnosis (p = 0.004), larger breast volume (p = 0.011), conventional fractionation (p < 0.001) and V > 107% were related to an increased risk of G2 – G3 skin reactions. Indeed, patients with no skin reaction (G0) or faint limited erythema were found to have a smaller V > 107% (3.9 cc ± 5.6 SD) in comparison to those who presented a true skin reaction (G2 – G3) whose V > 107% was, in average, 10.9 cc ± 8.7 SD. The difference is statistically significant (p < 0.001). No statistically significant difference was highlighted, on the contrary, for the maximum doses recorded in the ROIs.

**Table 5:** Univariate and multivariate analysis for G2-G3 radiation induced skin reactions in 200 patients

**Variables**	**Univariate analysis**		**Multivariate analysis**	
		**P**	**Odds ratio (95% CI)**	**P**
Chemotherapy	Yes vs No	0.054	1.137 (0.531-2.433)	0.741
Hormone Therapy	Yes vs No	0.401	1.230 (0.547-2.765)	0.616
Fractionation Schedule	CF vs HS	*< 0.001*	2.045 (0.996-4.197)	0.51
V > 107%	Mean ± SD	*< 0.001*	6.335 (3.192-12.577)	*< 0.001*
Age	Mean ± SD	*0.004*	0.973 (0.943-1.005)	0.101
Breast Volume	Mean ± SD	*0.011*	1.001 (1.000-1.002)	0.076

CI: confidence intervals; SD: standard deviation. CF: Conventional Fractionation;

HS**:** Hypofractionated Schedule.

At the multivariate analysis (Table 5) the only variable retaining significance was V > 107% (p < 0.001) while fractionation only showed a tendency towards significance in favour of HS vs CF. The RR for patients with V > 107% above the median value was 6 times higher than those with V > 107% below the median value.

**Figure 2:**
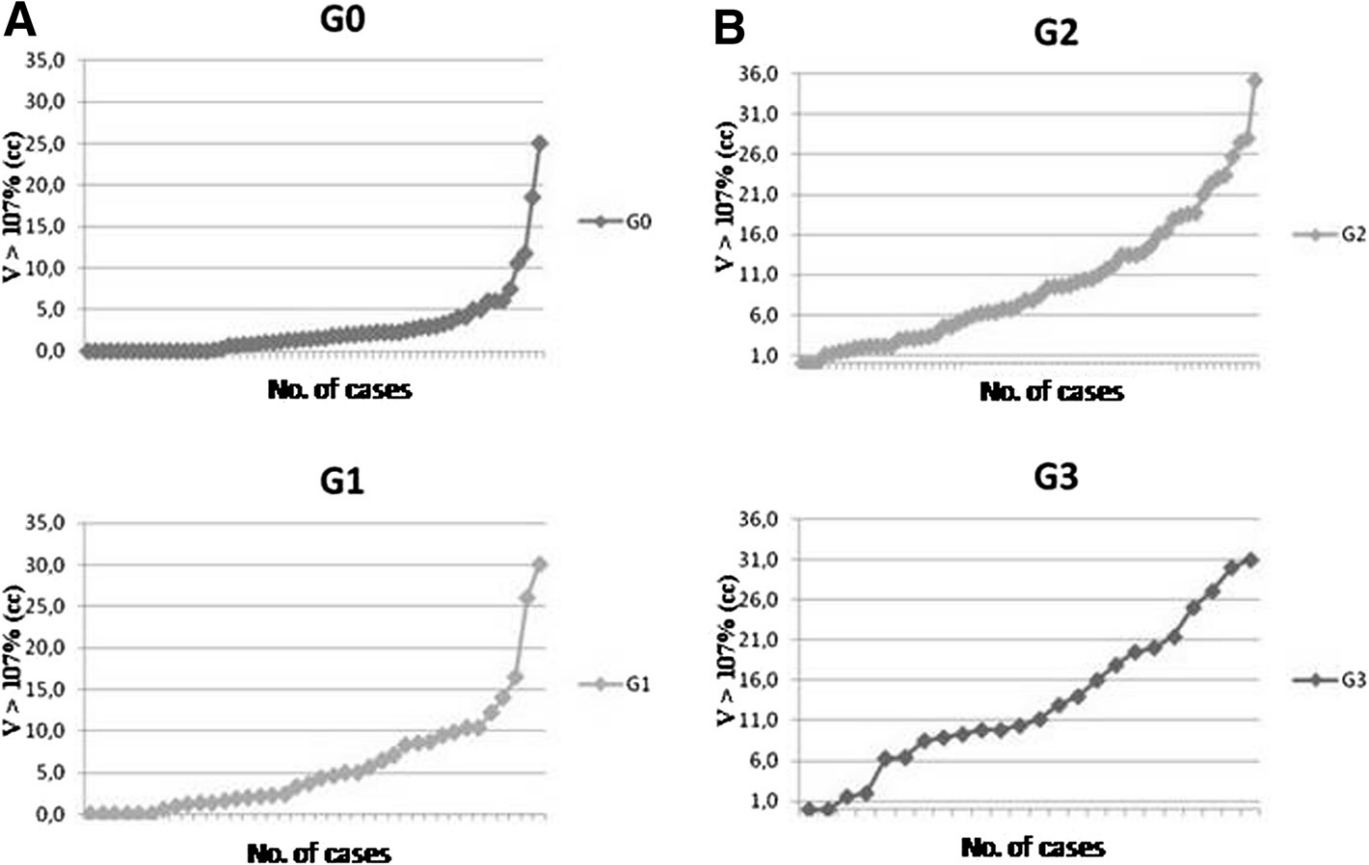
**Number of patients with any grade of skin reaction.** On the x axis is reported the number of patients showing any grade of skin toxicity and on the y axis the volume V > 107% (cc). **2A** - Patients with no or faint skin toxicity (G0-G1). **2B** - Patients with moderate to severe skin toxicity (G2-G3).

**Figure 3:**
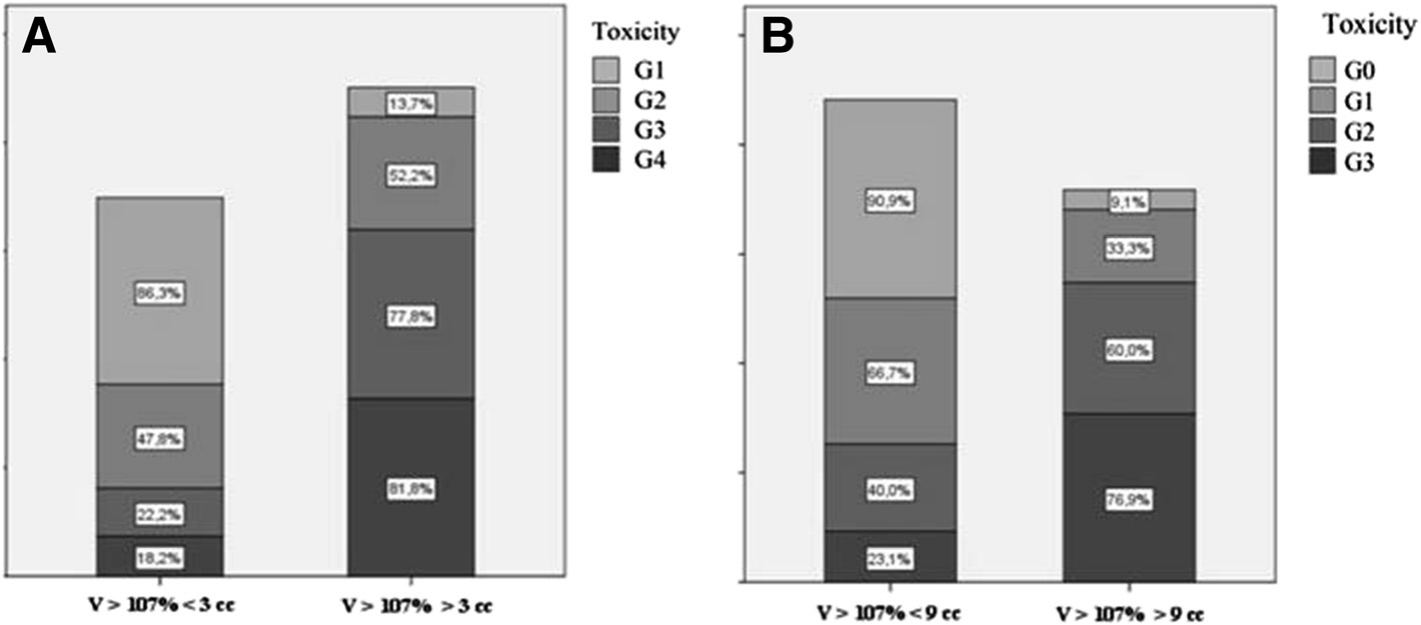
**Distribution of acute skin reactions in patients undergoing different fractionation and median V > 107 cut-off value. 3A** – Hypofractionation group where most of patients with G0- G1 reactions belong to the median cut-off V > 107% value < 3 cc. **3B** – Conventional fractionation group where most of patients with G0- G1 reactions belong to the median cut-off V > 107% value < 9 cc.

Figure 2 shows the number of patients with a volume receiving more than V > 107% by grade of skin reaction: hot spots are more represented among G2 – G3 patients. Plotting the data by the volumes receiving more than V > 107% for the HF and CF respectively we obtain a more detailed description of these results (Figure 3): patients with higher values of V > 107% are evidently more represented among those showing G2 and G3 skin reactions.

In this preliminary experience an average value distinguishing between high and low risk of developing mild to moderate or severe skin reaction has been found to be 3 cc for the HF patients and 9 cc for the CF ones (Figure 3). Volumes, in cubic centimetres, receiving more than 107% of the prescribed dose are larger among the CF group (mean 10 cc and median 9 cc) than among the HF patients (mean 5,7 cc and median 3 cc) (Table 2).

## Discussion

Acute toxicity after treatment for breast cancer is an issue which has been recently addressed in the literature [3,4] and which deserves consideration as severe skin toxicity can lead to temporary or permanent cessation of treatment causing pain and maybe permanent skin changes. After conservative surgery different fractionation schemes have been introduced in the treatment of breast cancer patients. With 5 and 10 years of follow-up the efficacy and cosmetic results of non conventional fractionation schemes have been proven, in comparison to the conventional ones, resulting in improved patient convenience and decreased resource utilization [13-15]. Patients included in the aforementioned randomized trials were predominantly treated with two dimensional planning techniques, non-uniform use of inhomogeneity corrections, and dose calculations limited to the central axis. In the past decade there have been substantial shifts in patterns of practice such that the 3D-CRT and IMRT became widespread and have been proven beneficial in improving dose homogeneity and reducing acute skin toxicity in randomized trials [16,17]. In a recent study Dorn and coll. have specifically examined dosimetric parameters and acute toxicity in patients with separation > 25 cm or large breast volume treated with HF. Their rates of acute toxicity (8.7%) compare favourably with those reported in other series of patients as well as with the data in our series (G3 13% and 7.5% respectively in the CF and HF patients) [8].

In our series (339 patients) the only factors associated with any grade of skin toxicity at a univariate analysis were age at diagnosis and fractionation. Breast volume, systemic treatments and other patient related factors did not show any influence on the occurrence of skin toxicity.

As concerns breast volume as a relevant factor related to skin toxicity contradictory data are available in the literature. Recently two papers [18,19] reach opposite conclusions. Corbin et al. [18] state that among obese and large breasted women (> 1500 cm^3^), in their experience, there was no increase in acute skin toxicity in the group undergoing hypofractionated radiation therapy. On the contrary Kraus-Tiefenbacher et al. [19] state that one of the factors associated with higher grade skin toxicity was, in their experience, the presence of larger breast volumes (range 402–4283 cc, median 946,9 cc). One possible explanation for these discrepancies may be that different criteria have been used to define what is considered “a large breast”. Freedman et al. [20] found a positive correlation between breast size and skin toxicity in larger breast volumes defined as > cup D. Vicini et al. [21] found that patients with breast volume > 1600 cc had more acute skin toxicity compared to those with smaller breast volumes (< 1000 cc). Another report [22] showed no G3 RTOG acute effect with breast volumes < 975 cc while patients with breast volumes greater then 1600 cc developed 59% RTOG G2 and 3% G3 erythema. As in our evaluation they measured breast volume in analogy to our method (manually contouring of breast target volume). The average breast volumes among our patients are smaller (718.7 cc, range between 188.6 cc and 2036.7 cc) than those reported in the previous papers. Even as concerns the role of systemic treatments before external beam radiation therapy on skin toxicity the data in the literature are contradictory. There are a few studies where a significant correlation between chemotherapy and increased skin toxicity was documented [21,22]. Different results have on the contrary been published [23,24] showing a trend to increase higher grade skin toxicity after chemotherapy.

Our statistical analysis was performed on 200 patients whose 3D-CRT were retrieved and revaluated to better define site and size of homogeneities of dose distribution and its relation with the site of the skin damage. We decided to revaluate the entire 3D plan, instead of relying only on the DVHs of the single patient which does not allow to locate the site of the dose inhomogeneities > 107% which we consider relevant to the development of acute skin toxicity. Among these patients age, type of fractionation (CF vs HS), breast volume and inhomogeneity of dose distribution (V > 107%) were statistically significant at the univariate analysis and correlated with skin reaction. On multivariate analysis, on the contrary, there was only an association between the heterogeneity parameter V > 107% and skin reaction which is not unexpected.

In two randomized trials [25,26] comparing IMRT to two-dimensional breast planning, improved homogeneity translated into reductions in acute toxicity supporting the need that an accurate evaluation of dose homogeneity, before treatment delivery, may reduce acute severe skin reactions.

IMRT may obtain a better dose distribution and a reduction of dose inhomogeneities, but it is more time consuming as concerns planning and delivery and it is, of course, more expensive. As the number of patients deserving a more complex treatment is limited to those at risk for severe skin damage, who are approximately less than 10% of all of the patients undergoing postoperative irradiation for breast cancer, IMRT should be considered after accurate examination of 3D dose distribution showing dose inhomogeneities greater than 107% of the prescribed dose.

As concerns age we underline that, at least in our series, it is unrelated to skin toxicity in spite of the significance obtained at univariate analysis (p = 0.001). At the beginning of our experience we selected, in fact, older patients to undergo treatment with the HF schedule and this selection has probably introduced a bias in the analysis.

Dosimetric analyses in the aforementioned randomised trials [13-15] were mainly performed at the central axis and therefore no information were available on the off-axis target volume and on site and size of hot spots and dose inhomogeneities. In this analysis off-axis heterogeneity was evaluated on the retrieved and revaluated 3-D plans allowing a clear definition of the site and size of the hot spots. Inhomogeneities were found to be significant and on multivariate analysis they were found to be the only factor retaining statistical significance [OR: 6.3 (95% CI: 3.192 - 12.577); p **<** 0.001]. The Odd Ratio for V > 107% is six times higher than that calculated for systemic therapies, age and breast volume, and three times higher than that related to fractionation supporting the strong relation between dose inhomogeneity and acute severe skin reaction.

The retrieval of the treatment plans allowed, moreover, an evaluation of the relation between the site of the hot spots and the occurrence of skin reaction. Hot spots were more frequent than skin reactions due to the fact that only inhomogeneities close to the skin surface were responsible of skin reactions which were consistent with the description of the site and size of moist desquamation reported in the clinical records. Hot spots deep seated in the breast parenchyma may probably contribute to the occurrence of different types of acute or late toxicities, but do not contribute, in our experience, to severe skin acute damage.

In our series skin toxicity appeared always immediately before or concomitant to the end of the treatment on the entire breast and always before the boost irradiation of the tumor bed excluding any role or responsibility of the boost as concerns the occurrence of the reaction.

Data from the literature and from our series show that dose inhomogeneities may be responsible for acute skin damage while the role of different patient or tumor related factors, other than breast volume, are not common to all of the reported experiences in the literature. Recently the attention has been focused on the fact that mismatch repair mechanisms may be involved in cellular response to RT and genetic polymorphisms [27] may be candidates for predicting acute radiosensitivity and we believe that further studies evaluating this hypothesis may contribute to a better comprehension of the steps leading to the occurrence of severe acute skin reactions.

## Conclusions

In conclusion:

1. The proportion of patients expected to experience high grade skin toxicity is on the order of 10 - 15% among HF patients and such rates compare favourably with those reported in our series (G3 13% and 7.5% respectively in the CF and HF patients) supporting the use of HF in the treatment of patients after BCS without any risk of increasing acute skin damage;

2. HF after BCS is safe and feasible and a shorter course of treatment in a broader range of patients may improve patient convenience and decrease resource utilization;

3. with the limits of a retrospective study our results suggests that dose inhomogeneties (V > 107%) have a significant impact on the occurrence of severe skin reactions as pointed out by Chen and coll. [7] too who demonstrated that a larger volume receiving > 53.9 Gy, within PTV (PTV - V107%), was a significant predictor of RT- induced skin toxicity;

4. deep seated dose inhomogeneities, which do not correlate with skin reaction, deserve particular attention to detect other types of side effects which have not yet been investigated and reported in the aforementioned randomized trials;

5. increasing interest is arising around the possibility that genetic polymorphisms may be candidates for predicting acute radiosensitivity and this aspect deserves attention and has to be investigated.

## Appendix

*n*: total number of fractions

*d*: size of fractions in Gy

*T*: overall time of treatment (days, with first day as D0)

*Tk*: onset (kick-off) time of repopulation in the tissue of interest: 21 days

*Tp*: potential doubling time of cancer repopulating cells: 3 days

*α*: radiosensitivity coefficient of non-recoverable damage: 0.35

## Competing interests

The authors have no financial disclosures or conflicts of interest to report.

## Authors’ contributions

Each of the co-authors has made important contributions to this manuscript. In particular: GT reviewed and analyzed the data, performed statistical analysis, created the figures, and drafted the manuscript. LDM reviewed and analyzed the data, performed statistical analysis, created the figures, drafted the manuscript and participated in the design of study. MDF participated in the design of study, interpretation and analysis of data. GT, LDM, RB, SC, DdC, DF, GI, DJ, PM, AM, EP, ST, BT were responsible in collection of dosimetric and clinical data. RS provided significant intellectual contribution, drafted and reviewed the manuscript. All authors read and approved the final manuscript.

## Pre-publication history

The pre-publication history for this paper can be accessed here:

http://www.biomedcentral.com/1471-2407/13/230/prepub
